# Validation of DNA probes for molecular cytogenetics by mapping onto immobilized circular DNA

**DOI:** 10.1186/1755-8166-1-28

**Published:** 2008-12-23

**Authors:** Karin M Greulich-Bode, Mei Wang, Andreas P Rhein, Jingly F Weier, Heinz-Ulli G Weier

**Affiliations:** 1 Division Genetics of Skin Carcinogenesis, German Cancer Research Center (DKFZ), Heidelberg, Germany; 2 Life Sciences Division, E.O. Lawrence Berkeley National Laboratory, University of California, Berkeley, CA, USA; 3 Klinikum Kaufbeuren, Dr.-Gutermann-Straße 2, D-87600 Kaufbeuren, Germany; 4 Reprogenetics, LLC, Oyster Point Blvd., South San Francisco, CA, USA

## Abstract

**Background:**

Fluorescence *in situ *hybridization (FISH) is a sensitive and rapid procedure to detect gene rearrangements in tumor cells using non-isotopically labeled DNA probes. Large insert recombinant DNA clones such as bacterial artificial chromosome (BAC) or P1/PAC clones have established themselves in recent years as preferred starting material for probe preparations due to their low rates of chimerism and ease of use. However, when developing probes for the quantitative analysis of rearrangements involving genomic intervals of less than 100 kb, careful probe selection and characterization are of paramount importance.

**Results:**

We describe a sensitive approach to quality control probe clones suspected of carrying deletions or for measuring clone overlap with near kilobase resolution. The method takes advantage of the fact that P1/PAC/BAC's can be isolated as circular DNA molecules, stretched out on glass slides and fine-mapped by multicolor hybridization with smaller probe molecules. Two examples demonstrate the application of this technique: mapping of a gene-specific ~6 kb plasmid onto an unusually small, ~55 kb circular P1 molecule and the determination of the extent of overlap between P1 molecules homologous to the human NF-κB2 locus.

**Conclusion:**

The relatively simple method presented here does not require specialized equipment and may thus find widespread applications in DNA probe preparation and characterization, the assembly of physical maps for model organisms or in studies on gene rearrangements.

## Background

Fluorescence *in situ *hybridization (FISH) has established itself in recent years as an independent method in high-resolution physical map assembly, often providing information that complements PCR-based STS contents mapping [1-5]. Initial characterization of clones by hybridization of non-isotopically labeled probes to metaphase chromosomes allows estimates of probe position within a 10–20 Mbp interval, often detecting clones that are chimeric or contain regions duplicated in the genome [6]. There is, however, a need to thoroughly characterize DNA probes for molecular cytogenetic studies, detect rearrangements within the probe such as deletions, duplications or inversions and anchor probes in a high resolution physical map.

Chromatin released from somatic cells by chemical or mechanical treatment provides a template onto which cloned DNA probes can be mapped to determine overlap or physical distance with even higher resolution [7-16]. The application of such techniques for the assembly of high-resolution physical maps, however, is quite inefficient because the genomic templates contain mostly non-target DNA sequences. In such template preparations, hybridization targets suitable for analysis are rare and, thus, hard to find in the useful area of a microscopic slide. The existence of duplicated genomic regions, gene families, or pseudogenes, which may produce additional hybridization domains further complicates the analysis of the hybridization pattern.

Novel techniques for mapping cloned probes onto DNA fragments enriched by pulsed field gel electrophoresis (PFGE) provide a much higher density of target DNA molecules [17,18]. At the same time, these techniques limit the complexity of the hybridization target so that some of the before-mentioned problems may be circumvented [11,19]. Mechanical deposition of DNA molecules, however, does not allow precise control of the extent of DNA stretching so that molecules are often stretched to various extents, and standards are needed for normalization and distance measurements [11,17,20]. Results from our and other laboratories suggest that stretching of DNA molecules bound to a solid substrate at one or both of their respective ends by the hydrodynamic action of a receding water meniscus termed 'molecular combing' [21] produces linear DNA molecules that are stretched homogeneously to approximately 2.3 kb/μm [18,22]. We have applied this procedure to prepare linear DNA molecules ('DNA fibers') ranging in size from 5–6 kb to more than 1000 kb for localization of restriction fragments, cloned DNA or cDNA sequences along the fibers or for measurement of physical distances between non-overlapping DNA molecules [3,4,23,24]. To emphasize the quantitative character of this optical mapping procedure comprised of molecular combing, FISH, and digital image analysis, we termed the process 'Quantitative DNA Fiber Mapping' or 'QDFM' [18,22,23].

Gene mapping and construction of high-resolution physical maps constitute major applications of QDFM in our laboratory. For these purposes, large insert DNA clones are obtained from the Dupont P1 library by screening [25,26]. As practiced previously [18], QDFM on the P1 clones required separation of high molecular weight DNA from the bacterial host, linearization of the circular P1 DNA molecules by restriction in the unique Not1 site within the P1 vector pSac BII, and PFGE purification of the linear P1 DNA molecules. The linearized P1 molecules were then bound to silanated glass slides, subjected to molecular combing, and used as mapping templates [23,27].

Occasionally, we encounter clones that are smaller than the 'standard' P1 clones of ~95 kb. These clones typically yield less DNA in our alkaline lysis protocol [18], and some of them do not digest properly with Not1. One of such small P1 clones is the clone RMC11P010 (registered with GDB as D11S3929), which contains the polymorphic marker D11S12 where an alleged lung cancer-specific suppressor gene resides [28,29].

In one QDFM-application we were interested in determining the location and extent of overlap between the plasmid probe pADJ762 [28] and RMC11P010. Initial fiber mapping on DNA molecules isolated from PFGE gels indicated that the linear molecules were randomly sheared DNA molecules, because the location of the vector sequence was observed randomly along the DNA fibers and attempts to map a 6 kb plasmid onto this P1 clone RMC11P010 were complicated by the variability of the DNA molecules.

In another case, we intended to determine the overlap between two P1 clones '3012' and '3015'. Linearization of P1 DNA by Not1 digestion would have separated part of the insert from the vector and led to loss of mapping information as a result of the presence of one or more Not1 sites in the insert. Therefore, we decided to investigate the deposition of circular, uncut P1 DNA onto the solid substrate and performed molecular combing and physical DNA fiber mapping on circular DNA molecules.

## Results and Discussion

### Clone selection, validation, Not1 restriction digestion and PFGE

A large insert, human genomic DNA probe for FISH ('RMC11P010') was isolated by PCR screening of the Dupont P1 library [23,26]. By FISH, this clone was mapped to the correct chromosome band (11p15.5) (data not shown).

**Figure 1:**
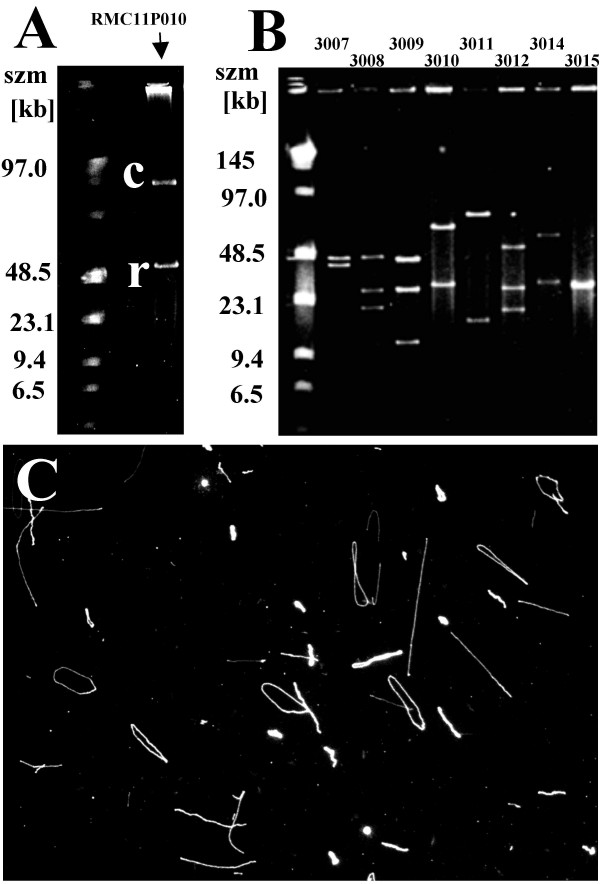
**P1 DNA molecules of different size**. A) PFGE-analysis of the P1 clone RMC11P010. After incubation with Not1, pulse field gel electrophoresis showed circular DNA (**c**) and a band containing relaxed molecules (**r**) corresponding to a size of approximately 55 kb. The size marker lane ('szm', left) contains 500 ng of low range PFGE marker. B) Gel electrophoretic analysis of P1 clones containing part or all of the human NF-κB2 gene. The DNA from individual clones were digested with Not1 and separated by PFGE. All clones produced two or more restriction fragments indicating the presence of at least one Not1 site within the P1 insert. The size marker lane (szm) contains 500 ng of low range PFGE marker. C) Staining of gel-purified 'circular' P1 DNA molecules with YOYO-1 (Molecular Probes) reveals circular and randomly broken DNA molecules on the slide. Most of the DNA molecules shown here in the liquid phase prior to molecular combing are bound to the substrate with one end or via nicks in circular molecule.

The P1 clone RMC11P010 presented several problems: DNA yields from our alkaline lysis protocol were unusually low and the isolated DNA could not be digested with Not1. The PFGE analysis of the P1 lysate showed a band containing circular DNA molecules (which have lower electrophoretic mobility than the linear molecules [22]) ('c' in Fig. 1A) in addition to the linear P1 molecules sheared during preparation indicating an unusually small P1 clone of ~55 kb ('r', Fig. 1A). DNA fiber mapping indicated that the linear molecules were randomly broken DNA molecules because the location of the vector sequence was observed randomly along the DNA fibers (data not shown). Furthermore, the vector part of the DNA fibers appeared shorter than usual (~15 kb instead of ~17 kb, QDFM result, data not shown) suggesting deletion of the Not1 site. When the band containing 'circular' DNA purified from a PFGE slice was stained with YOYO-1 (Molecular Probes, Eugene, OR), both circular and randomly broken linear DNA molecules were observed (Fig. 1C).

While deletion of the unique Not1 site in the P1 vector has been complicating QDFM, the presence of one or more Not1 sites within the insert leads to undesirable fragmentation as shown in the following example. The DNAs from eight P1 clones that contain part of the human NF-κB2 gene (also known as "lyt-10") [30] were digested with Not1 and separated by PFGE (Fig. 1B). Most of the clones produced several restriction fragments indicating the presence of one or more Not1 sites within the P1 insert (Fig. 1B). On the other hand, FISH experiments using metaphase chromosome spreads confirmed that all clones mapped to chromosome 10q24 as expected (data not shown). Among the P1's, clones '3012' and '3015' showed positive PCR amplification with primers for either the 5'- or 3'-end of NF-κB2 and were selected for QDFM analysis of circular DNA. Restriction enzyme analysis after complete Not1 digest had suggested that both clones have one restriction fragment of ~27 kb in common which was not shared with five of the other of NF-κB2 clones (Fig. 1B).

### Quantitative DNA Fiber Mapping

To circumvent the obstacles of vector-Not1 site deletion or presence of additional Not1 site(s) in the insert, we decided to investigate the possibility of mapping directly onto circular molecules. Two slightly different preparative methods were used to obtain the DNA fibers: In the first approach, we isolated non-digested circular DNA molecules of clone RMC11P010 after incubation with Not1 and PFGE, and then mapped a homologous plasmid directly onto this P1 molecule. In the second set of experiments, we skipped the restriction enzyme digestion and PFGE purification steps and directly immobilized P1 clone '3012' DNA obtained by an alkaline lysis procedure that isolates only closed circular DNA molecules. Another circular P1 clone '3015' isolated by the same approach was labeled differentially and mapped onto '3012' to determine their overlap.

**Figure 2:**
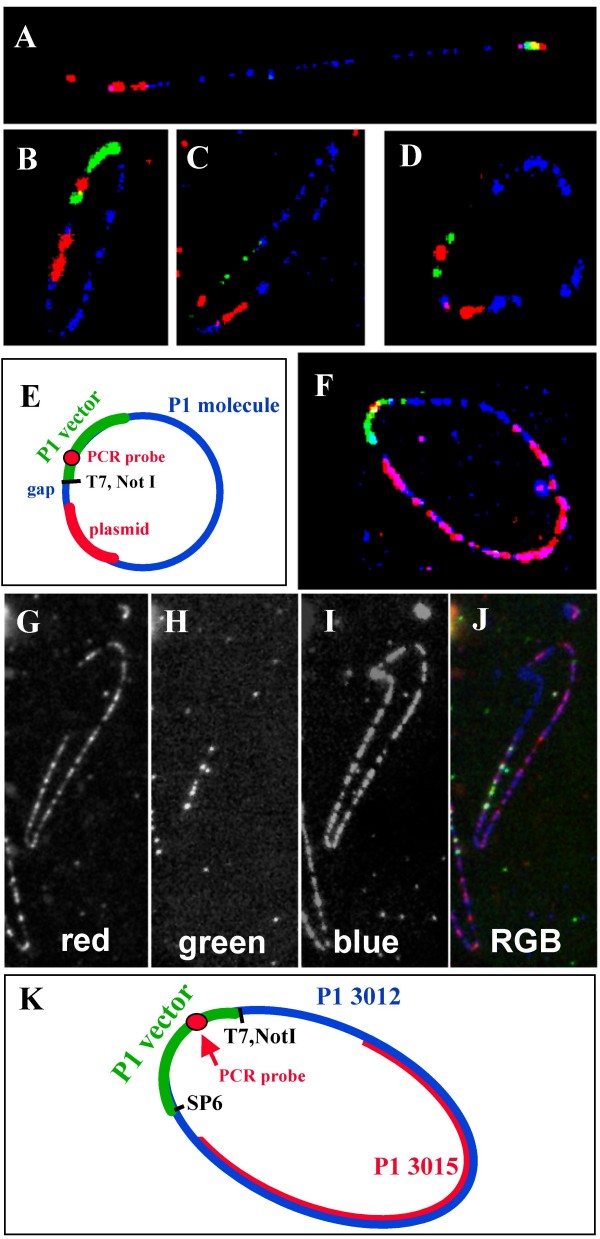
**High resolution physical mapping by QDFM to linear and circular DNA molecules**. A)-D) Isolated molecules of RMC11P010 were hybridized with plasmid probe pADJ762 (red) and a probe DNA specific for the P1 vector pSac BII (green). The entire P1 DNA molecules were counterstained by hybridization of random-primed RMC11P010 DNA (blue). A PCR generated ~1400 bp probe that maps in the pAd10SacBII vector near the T7 promoter site was included in the hybridization mix to show the vector orientation. A linear DNA molecule is presented in A). E) Schematic representation of the hybridization result shown in B-D). The approximate location of the Not1 site in a 'normal' P1 is indicated. F) Analysis of the overlap between two linked P1 clones ('3012', '3015') that contain one or more Not1 sites in the insert. The labeling and detection scheme is described in Table 3. G)-J) The red, green and blue images of hybridization signals along a single, closed circular DNA molecule ('3012') as well as the RGB image (J) generated by superposition of images G)-I). K) Schematic diagram showing the overlap between clones '3012' and '3015' and the location of P1-vector-specific probes.

Analysis of clone RMC11P010 by QDFM showed that a) the vector part of this P1 clone is relatively small, only ~15 kb long, b) the human genomic insert amounts to ~40 kb, which is less than the average P1 insert size of ~80 kb [25,26], and c) the plasmid clone pADJ762 and RMC11P010 overlap about 5.9 kb near the end of the insert at the T7 promoter (Fig. 2A–D). A schematic diagram of the various components of the hybridization: P1 fiber, P1 vector, vector specific PCR fragment, the plasmid clone, the gap between plasmid and P1, and their overlap is shown on Fig. 2E.

By applying our regular protocol for molecular combing, the binding efficiency of circular DNA molecules to the derivatized slides [27] was similar to preparations of linearized DNA. Applying the circular mapping scheme to measurement of the overlap between P1 and plasmid clone, our analysis indicated a gap of 3 kb +/- 1.2 kb (N = 8) between the T7 promoter of the P1 vector and the plasmid DNA probe. The results confirmed that the region homologous to pADJ762 is fully contained in P1 clone RMC11P010.

In our P1-on-to-P1 hybridizations, the biotinylated probe prepared for the immobilized DNA molecules allowed us to determine the density and quality of DNA fibers on slides. A typical slide prepared with PFGE-purified circular '3012' P1 molecules carried dozens, if not hundreds, of high quality DNA molecules. The P1 vector part was readily discernable due to binding of the vector-specific FITC-labeled probes (Fig. 2F). In this dual-P1 clone mapping, the red-visualized clone '3015' overlaps with the fiber (blue) for approximately 65%, with a large gap near the T7 promoter and a smaller gap near SP6 end of the P1 vector (Fig. 2F–K). The analysis of individual molecules in triplicate revealed an average value of 68.17 +/- 7.37 kb (mean +/- standard deviation) for the overlap between the P1 clones, and an average size of the counterstained vector part of 15.88 +/- 1.78 kb (Table 1). Given the small number of molecules analyzed (N = 7, Table 1) and our simple experimental set-up, a relative standard deviation of slightly more than 10% is considered a sign of excellent reproducibility.

**Table 1:** Measurements of clone overlap

	vector		overlap		unique		total molecule	
**Image**	pixel	kb	3015 (red)	kb	3012 (blue)	kb	pixel	kb(calc)

066.05.2	57.33	14.65	225.82	66.96	86.48	25.64	107.26	94.46

	56.47	14.43	224.75	67.66	86.06	25.91	108.00	93.86

	56.50	14.44	226.17	68.05	86.02	25.88	108.37	94.22

066.06.1	59.67	15.25	273.86	78.02	136.69	38.94	132.21	120.17

	60.76	15.53	273.85	76.62	136.52	38.20	130.34	120.40

	59.67	15.25	274.61	78.24	136.52	38.89	132.38	120.32

066.07.1	73.97	18.90	291.71	67.04	127.09	29.21	115.15	125.93

	73.64	18.82	292.46	67.52	127.60	29.46	115.79	126.17

	74.38	19.01	292.48	66.85	127.74	29.20	115.05	126.40

066.09.1	66.10	16.89	305.03	78.45	147.42	37.91	133.26	132.52

	65.03	16.62	306.61	80.15	149.41	39.06	135.83	133.16

	65.26	16.68	306.00	79.71	148.55	38.70	135.09	132.84

066.11.1	68.06	17.39	255.43	63.80	105.66	26.39	107.59	109.67

	68.05	17.39	255.34	63.79	104.11	26.01	107.19	109.25

	68.17	17.42	255.79	63.79	105.82	26.39	107.60	109.83

066.17.1	53.91	13.78	182.75	57.63	77.20	24.34	95.75	80.21

	53.74	13.73	182.24	57.65	76.99	24.35	95.74	79.98

	52.46	13.41	182.83	59.25	76.48	24.78	97.44	79.67

066.19.2	57.31	14.65	214.69	63.68	84.49	25.06	103.39	91.10

	56.08	14.33	212.95	64.55	83.69	25.37	104.25	90.14

	58.64	14.99	214.31	62.13	83.83	24.30	101.42	91.18

								

average		15.88		68.17		29.71		

std. deviation		1.78		7.37		5.96		

Quantitative DNA Fiber Mapping (QDFM) has proven to be a highly useful technique for localization of DNA sequences within larger genomic intervals as well as for high resolution physical map assembly [3,4,23]. While the exact mechanism of coupling circular DNA molecules to APS-derivatized slides remains unknown, we attribute the observed binding to single strand nicks in the circular DNA molecule. We also prepared PFGE-purified circular DNA molecules from a PAC clone (11C11, ~125 kb) that contains a Not1 site within the insert and found similar good binding of DNA circles in the presence of linear molecules (data not shown). Circular DNA can be purified from P1, PAC or BAC clones by simple techniques and mapping onto DNA circles would save time-consuming PFGE steps and minimize loss of material during preparation. Further development of this technique will also circumvent problems associated with Not1-linearization such as digestion within the insert or, as in the case of clone RMC11P10, absence of the unique Not1 site due to partial deletion of P1 vector DNA.

The mechanisms of binding of purified DNA molecules to amino-silanated glass is not yet fully understood, but studies using linear double-stranded DNA molecules such as phage DNA or yeast artificial chromosomes showed preferential binding of the ends of the DNA molecules to the solid substrate [17,20]. We therefore assumed a very low binding efficiency for circular DNA and a somewhat higher binding efficiency when DNA circles were nicked on one strand.

Molecular combing stretches DNA molecules to approximately 2.3 kb/μm [21,23]. At this scale, a closed circular DNA molecule of 55 kb is expected to have a perimeter of 23.9 μm or, when deposited as a perfect circle, a diameter of 7.6 μm. Similarly, a DNA molecule of 95 kb, the average size of most P1 DNA clones, would produce a circle with a diameter of about 13 μm. Resolution of circles of this size is well within the range of visible light microscopy and FISH signals along the perimeter should thus be easy to map by image analysis.

The hydrodynamic force did not stretch circular DNA molecules as well as linear molecules and, following hybridization, circular DNA molecules were found stretched to various extents. This required the normalization of distance measurements using the known size of the plasmid vector probe (5.5 kb) or the P1 vector (17 kb) as internal standard. While this appears to be a weakness of mapping onto circular DNA molecules, slides always contained a number of double-stranded, randomly broken DNA molecules, which were found stretched to the expected length of about 2.3 kb/μm [23].

The present study was initiated to explore the utility of QDFM for validation of recombinant DNA molecules used in physical mapping and cytogenetic studies, and considering their suitability as probes in FISH experiments, in tumor research. This study explores the types of template DNA molecules preparation methods that can be used for physical mapping. Problems associated with the linearization of circular DNA molecules for QDFM and the subsequent binding of fragments are addressed in the present paper. Certain DNA cloning vectors facilitate the preparation of DNA fibers, because they contain a single binding site for a rare-cutting restriction enzyme, such as the unique Not1 site in the P1 vector. However, the use of Not1 for linearization of DNA molecules is not without its limitations: some vectors such as the BAC's contain not one, but two Not1 binding sites intended to allow the convenient excision of the entire cloning cassette. On the other hand, CpG islands around genes may give rise to one or several Not1 sites, as we found it in the case of the NF-κB2 genes and the genomic P1 clones ('3012', '3015') described in this paper. Thus, if BAC clones need to be characterized without separating the vector part from the insert, a method other than Not1 digestion needs to be applied to prepare the linear DNA molecules.

A second problem addressed in the present paper is related to insert rearrangements and, in particular, deletions in large insert DNA clones. Not only do deletions complicate the assembly of high resolution physical maps, but the effect such deletions may be detrimental to molecular cytogenetic efforts characterizing small gene amplifications or deletions with FISH. The results reported here demonstrate convincingly that relatively simple modifications to the standard QDFM protocol allow addressing and solving the before-mentioned problems.

Thus, the results presented here extend the useful range of QDFM to circular P1, PAC and BAC DNA molecules, thereby circumventing problems related to linearization of phagemid DNA by enzymatic digestion.

## Conclusion

Accurate characterization of large DNA Quantitative DNA Fiber Mapping (QDFM) has demonstrated its utility in a number of studies, when unambiguous information about clone inserts, length, orientation and overlap was crucial for the construction of high resolution physical maps. In QDFM, a small amount of DNA isolated from recombinant DNA clones is linearized, bound with one or both ends to a solid substrate (e.g., a glass slide or sheet of freshly cleaved mica) and stretched by the tension force at the air-water interface of a receding meniscus.

Circular DNA molecules, prepared routinely from plasmid, P1 or BAC clones by DNA isolation protocols like the alkaline lysis protocol described above, can be bound to an APS-derivatized glass surface. Details of the binding mechanism are yet unknown, but it appears to be mediated through nicks in the DNA molecules introduced during the isolation process. While such nick-mediated, multi-point DNA molecule attachments help to 'tackle down' entire molecules, it does compromise subsequent stretching. But this approach is not as limited as it may appear. Typical DNA isolation protocols exert significant shearing forces onto the high molecular weight DNA molecules, and the resulting pool of DNA molecules is usually comprised of super-coiled, closed circular and randomly broken molecules, a fact we exploited in our experiments.

Two sets of mapping experiments described in this paper demonstrate that a more-or-less heterogeneous population of P1 molecules can be prepared with standard laboratory tools and procedures. Mixtures of circular and randomly linearized molecules then allow QDFM mapping experiments that show recombinant DNA inserts in the entirety (circles), thus circumventing problems related to unintentional molecule fragmentation or DNA loss. Although stretched to varying degrees, so that the universal QDFM conversion factor of 2.3 kb/μm no longer applies, insert parts of these DNA circles can be characterized by normalizing measurements using the known size of the vector part.

## Methods

### PCR screening of a large recombinant library

The cloned probe pADJ762 (marker D11S12) contains a DNA fragment that detects multiple restriction fragment length polymorphisms in human genomic DNA [28]. Studies of lung cancer specimen demonstrated loss of heterozygosity (LOH) in more than 70% of informative cases, pointing to the vicinity of D11S12 as candidate location of a lung cancer-specific tumor suppresser gene [29,31]. However, southern blot analysis did not allow to determine whether LOH is a consequence of allelic deletion or other mechanisms of reduction to hemizygosity such as homologous recombination. To facilitate fluorescence in situ hybridization (FISH) – studies that complement the more conventional LOH studies, we had one end of the insert of pADJ762 sequenced and prepared oligonucleotides suitable for in vitro DNA amplification using PCR.

Custom oligonucleotides (Genset, Inc., San Diego, CA) were used for PCR-screening of the Dupont P1 library [25] and for STS content mapping of selected clones [18]. The insert of plasmid clone pADJ762 containing D11S12 [28] was sequenced from one end using an M13 forward primer. For screening of the human genomic P1 library [25], a 120 bp stretch of DNA sequence was selected as amplification target using primers F-D11S12 and R-D11S12 (Table 2). The PCR with F-D11S12 and R-D11S12 isolated a single P1 clone of approximately 55 kb (RMC11P010, position 111H8 in the Dupont P1 library) [31]. The PCR parameters on a Perkin Elmer 9600 instrument specified denaturation at 94°C for 1 min followed by primer annealing and extension at 53°C for 1 min and 72°C for 2 min, respectively.

**Table 2:** Oligonucleotides used as PCR primers

Primer	Primer Sequence	Position in vector (v) or insert (i)	Product (bp)
F-D11S12	5'-CCTGATTAGAGGTCTTTCAG-3'	n.a. (i)	
R-D11S12	5'-TGGGGCTTAAAGAATGGATC-3'	n.a. (i)	120

F- NF-κB22	5'-CCCAGAGACATGGAGAGTTGCTAC-3	242–265 (i) [33]	
R- NF-κB22	5'-TGTTCCACAGTCACCAGGTAGG-3'	363–384 (i) [33]	143
F-LYT10	5'-GCCTCAGGTGCACTGACCTG-3'	2759–2777 (i) [30]	
R-LYT10	5'-ATTTGTCCCAACTGAGGGGT-3'	2858–2877 (i) [30]	120
F1-P1	5'-TACCCCATTTAGGACCACCCAC-3'	10054–10075 (v) [34]	
B1-P1	5'-CAGCCGAAGCCATTAAGGTTC-3'	11477–11497 (v) [34]	1444

For screening of the P1 library with a probe for NF-κB p50/p105 (i.e. NF-κB2), we selected a cDNA clone that contained ~550 bp including the rel homology domain [32]. For sequence tagged sites (STS) content mapping of isolated P1 clones, we had 2 pairs of oligonucleotides synthesized (Table 2). The set F1-/B1- NFκB22 flanks a stretch of 143 bp in the 5'-end of the human NF-κB2 gene including the 5'-UTR and the DNA coding for first 5 amino acids [33]. A second set of primers (F-/R-lyt10) binds near the 3'-end of the gene and amplifies a 120 bp product including the DNA coding for the last 4 amino acids, the stop codon and the proximal part of the 3'-UTR [30]. To provide a reference signal within the P1 vector part for determination of the insert orientation, we PCR-amplified a 1444 bp DNA fragment from the P1 vector with primers F1-/B1-P1 as described [34].

### DNA fiber preparation

High molecular weight DNA from cosmid, BAC/PAC or P1 clones is routinely isolated in our laboratory using an alkaline lysis protocol [23,35,36]. This included the P1 clone RMC11P10 [31] and the clones '3012' and '3015' used in the experiment described here. Following DNA extraction and precipitation clones '3012' and '3015' were used directly for fiber preparation. The DNA isolated from overnight cultures of clone RMC10P10 DNA was digested with Not1, size-separated by PFGE in multiple lanes and bands corresponding to undigested circular DNA were excised. Subsequent purification of DNA, glass slide cleaning, silane modification, application of the DNA molecules to the slides, and molecular combing were performed as described [22,27,37-39].

### Restriction digestion and PFGE

Prior to sizing by pulsed field electrophoresis, approximately 1 μg of P1 DNA molecules were incubated with 10 units Not1 for 90 min at 37°C in reaction buffer provided by the manufacturer (New England Biolabs (NEB), Beverly, MA). The DNA was then loaded on a 1.0% low melting point agarose gel. A size marker lane ('smz', Fig. 1A, B) contained 500 ng of low range PFGE marker (NEB). Electrophoresis proceeded for 15 hrs using a BioRad CHEF Mapper (BioRad, Hercules, CA) as described [23]. The band containing the circular P1 DNA ('c' in Fig. 1A) was then excised from the gel, the slice was equilibrated with agarase buffer and the gel slice was digested with agarase as recommended by the supplier (NEB) [27].

### Probe preparation and labeling

High molecular weight DNA probes from clones 111H8, '3012', and '3015' of a human genomic P1 library [25,26] were generated with DNA prepared from overnight cultures by an alkaline lysis protocol [23]. The DNA from plasmid clone pADJ762 and the recombinant cloning vector pAd10SacBII (~17 kb, Genome Systems, Inc., St. Louis, MO) were prepared by alkaline lysis followed by phenol:chloroform extraction [40]. For the P1 vector-specific DNA probes, a primer pair (F1-P1/B1-P1, Genset Corp., La Jolla, CA) was used in *in vitro *DNA amplification reactions to produce a 1.44 kb DNA fragment. The sequences of these primers, position along the respective vector and the PCR product size are included in Table 2.

The P1 clone pAd10SacBII containing vector DNA without insert was linearized with *Bam*H I before serving as PCR template for generating P1 vector-specific probe [22,34]. Two hundred microliters of PCR buffer [23] were used for each reaction; and the PCR parameters were 30 cycles of 94°C (1 min), 53°C (1 min), and 72°C (2 min) followed by a 10 min at 72°C for elongation. After validation by agarose gels, the PCR products were separated from the overlaying mineral oil by chloroform extraction. The DNA was then precipitated with 2 volumes of ethanol followed by a wash with 70% ethanol and air drying. All DNA concentrations were determined with Hoechst fluorometry (TKO100, Amersham Pharmacia Biotech, Piscataway, NJ). Four hundred and twenty nanograms of DNA were used for 50 μl labeling reactions. Labeling was performed by random priming reactions using a commercial kit (BioPrime kit, Invitrogen, Gaithersburg, MD) incorporating biotin (bio)-14-dCTP. To label with digoxigenin (dig)-11-dUTP (Roche Diagnostics, Indianapolis, IN) or FITC-12-dUTP (Roche), we used a previously published random priming protocol [18,37,38]. The labeling and hybridization detection schemes for the probes are summarized in Table 3.

**Table 3:** Probe labeling and detection scheme

**DNA fiber**	**Fiber probe**	**Insert probe**	**Vector-specific probe**
P1 clone 111H8	P1 clone 111H8-biotin (blue)	pADJ762-dig (red)	P1 vector pAd10SacBII-FITC (green) P1-F1/P1-B1-dig (red)

P1 #3012	P1 #3012-biotin (blue)	P1 #3015-dig (red)	P1 vector pAd10SacBII-FITC (green) P1-F1/P1-B1-dig (red)

### FISH and image analysis

To map the plasmid pADJ762 onto P1 clone RMC11P010, we combined a biotinylated probe prepared from RMC11P010 DNA, an FITC-labeled probe from the P1 vector pAd10SacBII [25,41], a digoxigenin (dig.)-labeled vector-specific PCR product of ~1400 bp that hybridizes to the P1 vector DNA near the Not1 site, and a dig.-labeled DNA probe prepared from plasmid pADJ762 in the hybridization mixture. For determination of overlap between P1 clones '3012' and '3015', the hybridization mixture contained the biotinylated probe prepared from P1 clone '3012' and dig.-labeled probe from P1 '3015' in addition to the FITC-labeled P1 vector probe and dig.-labeled vector-specific PCR product.

Biotinylated probes were detected in blue using avidin-AMCA (Vector Labs, Burlingame, CA) and signals amplified with biotinylated goat-anti-avidin (Vector) followed by a second layer of avidin-AMCA. Digoxigenin-labeled probes were detected with rhodamine-conjugated sheep antibody against digoxigenin (Roche) [23], then amplified with Texas Red-labeled rabbit antibody against sheep IgG (Vector). Detection of FITC labeled probes was performed with a mouse anti-FITC antibody (DAKO, Carpintera, CA) followed by incubation with an FITC-conjugated horse-anti-mouse antibody (Vector). Slides were washed twice in 2 × SSC and mounted in 1% p-phenylenediamine (PPD, Sigma Chemicals, St. Louis, MO) antifade solution in glycerol for microscopic inspection.

Images were acquired on a quantitative image processing system based on a Zeiss Axioskope fluorescence microscope equipped with 63x, 1.25 NA and 40x, 1.3 NA oil objectives, a Photometrics cooled CCD camera, multiband pass filters for simultaneous observation of FITC, Texas Red, or AMCA/DAPI (Chroma Technology, Brattleboro, VT), and a SUN SPARC workstation (Sun Microsystems, Inc., Mountain View, CA). Images were recorded on the SUN system, converted to 24-bit tiff format images, imported to a PC-based system, and processed with PhotoShop 4.0 (Adobe Systems Inc., San Jose, CA) and PowerPoint 97 (Microsoft Corporation, Redmond, WA).

All distances along the DNA fibers were measured in pixels, and converted into kb using the known pixel spacing of the camera and the factor for fully extended DNA of 2.3 kb/μm [23].

## Abbreviations

APS: aminopropyl tri-ethoxysilane; PCR: polymerase chain reaction; PFGE: pulsed field gel electrophoresis; QDFM: Quantitative DNA Fiber Mapping; SSC: standard sodium citrate; STS: Sequence tagged site; UTR: untranslated region

## Competing interests

The authors declare that they have no competing interests.

## Authors' contributions

All of the authors have directly participated in the planning, execution, and analysis of the study and resulting paper. Specifically, MW and KG-B carried out the DNA fiber mapping experiment, AR and JW conducted the PFGE experiments, MW, KG-B, JF and HW analyzed the fibre mapping images, HW was in charge of probe acquisition and project coordination. All authors reviewed results, were involved in the preparation of the manuscript and read and approved the final manuscript.

## Disclamer

This document was prepared as an account of work sponsored by the United States Government. While this document is believed to contain correct information, neither the United States Government nor any agency thereof, nor The Regents of the University of California, nor any of their employees, makes any warranty, express or implied, or assumes any legal responsibility for the accuracy, completeness, or usefulness of any information, apparatus, product, or process disclosed, or represents that its use would not infringe privately owned rights. Reference herein to any specific commercial product, process, or service by its trade name, trademark, manufacturer, or otherwise, does not necessarily constitute or imply its endorsement, recommendation, or favoring by the United States Government or any agency thereof, or The Regents of the University of California. The views and opinions of authors expressed herein do not necessarily state or reflect those of the United States Government or any agency thereof or The Regents of the University of California.

## References

[B1] Smith DJ, Stevens ME, Sudanagunta SP, Bronson RT, Makhinson M, Watabe AM, O'Dell TJ, Fung J, Weier HU, Cheng JF, Rubin EM (1997). Functional screening of 2 Mb of human chromosome 21q22.2 in transgenic mice implicates minibrain in learning defects associated with Down syndrome. Nat Genet.

[B2] Cheng JF, Weier HU, Fox CF, Conner TH (1997). Approaches to High Resolution Physical Mapping of the Human Genome. Biotechnology International.

[B3] Duell T, Wang M, Wu J, Kim UJ, Weier HU (1997). High-resolution physical map of the immunoglobulin lambda variant gene cluster assembled by quantitative DNA fiber mapping. Genomics.

[B4] Duell T, Nielsen LB, Jones A, Young SG, Weier HU (1997). Construction of two near-kilobase resolution restriction maps of the 5' regulatory region of the human apolipoprotein B gene by quantitative DNA fiber mapping (QDFM). Cytogenet Cell Genet.

[B5] Vissers LE, de Vries BB, Osoegawa K, Janssen IM, Feuth T, Choy CO, Straatman H, Vliet W van der, Huys EH, van Rijk A, Smeets D, van Ravenswaaij-Arts CM, Knoers NV, Burgt I van der, de Jong PJ, Brunner HG, van Kessel AG, Schoenmakers EF, Veltman JA (2003). Array-based comparative genomic hybridization for the genomewide detection of submicroscopic chromosomal abnormalities. Am J Hum Genet.

[B6] Selleri L, Eubanks JH, Giovannini M, Hermanson GG, Romo A, Djabali M, Maurer S, McElligott DL, Smith MW, Evans GA (1992). Detection and characterization of "chimeric" yeast artificial chromosome clones by fluorescent in situ suppression hybridization. Genomics.

[B7] Heng HH, Squire J, Tsui LC (1992). High-resolution mapping of mammalian genes by in situ hybridization to free chromatin. Proc Natl Acad Sci USA.

[B8] Wiegant J, Kalle W, Mullenders L, Brookes S, Hoovers JM, Dauwerse JG, van Ommen GJ, Raap AK (1992). High-resolution in situ hybridization using DNA halo preparations. Hum Mol Genet.

[B9] Lawrence JB, Carter KC, Gerdes MJ (1992). Extending the capabilities of interphase chromatin mapping. Nat Genet.

[B10] Haaf T, Willard HF (1992). Organization, polymorphism, and molecular cytogenetics of chromosome-specific alpha-satellite DNA from the centromere of chromosome 2. Genomics.

[B11] Parra I, Windle B (1993). High resolution visual mapping of stretched DNA by fluorescent hybridization. Nat Genet.

[B12] Heng HH, Tsui LC (1994). Free chromatin mapping by FISH. Methods Mol Biol.

[B13] Lavania UC, Yamamoto M, Mukai Y (2003). Extended chromatin and DNA fibers from active plant nuclei for high-resolution FISH. J Histochem Cytochem.

[B14] Li L, Yang J, Tong Q, Zhao L, Song Y (2005). A novel approach to prepare extended DNA fibers in plants. Cytometry A.

[B15] Yamamoto M, Mukai Y (2005). High-resolution physical mapping of the secalin-1 locus of rye on extended DNA fibers. Cytogenet Genome Res.

[B16] Jiang J, Gill BS (2006). Current status and the future of fluorescence in situ hybridization (FISH) in plant genome research. Genome.

[B17] Heiskanen M, Karhu R, Hellsten E, Peltonen L, Kallioniemi OP, Palotie A (1994). High resolution mapping using fluorescence in situ hybridization to extended DNA fibers prepared from agarose-embedded cells. Biotechniques.

[B18] Weier HU, Rhein AP, Shadravan F, Collins C, Polikoff D (1995). Rapid physical mapping of the human trk protooncogene (NTRK1) to human chromosome 1q21-q22 by P1 clone selection, fluorescence in situ hybridization (FISH), and computer-assisted microscopy. Genomics.

[B19] Tocharoentanaphol C, Cremer M, Schrock E, Blonden L, Kilian K, Cremer T, Ried T (1994). Multicolor fluorescence in situ hybridization on metaphase chromosomes and interphase Halo-preparations using cosmid and YAC clones for the simultaneous high resolution mapping of deletions in the dystrophin gene. Hum Genet.

[B20] Florijn RJ, Bonden LA, Vrolijk H, Wiegant J, Vaandrager JW, Baas F, den Dunnen JT, Tanke HJ, van Ommen GJ, Raap AK (1995). High-resolution DNA Fiber-FISH for genomic DNA mapping and colour bar-coding of large genes. Hum Mol Genet.

[B21] Bensimon A, Simon A, Chiffaudel A, Croquette V, Heslot F, Bensimon D (1994). Alignment and sensitive detection of DNA by a moving interface. Science.

[B22] Wang M, Duell T, Gray JW, Weier HU (1996). High sensitivity, high resolution physical mapping by fluorescence in situ hybridization on to individual straightened DNA molecules. Bioimaging.

[B23] Weier HU, Wang M, Mullikin JC, Zhu Y, Cheng JF, Greulich KM, Bensimon A, Gray JW (1995). Quantitative DNA fiber mapping. Hum Mol Genet.

[B24] Admire A, Shanks L, Danzl N, Wang M, Weier U, Stevens W, Hunt E, Weinert T (2006). Cycles of chromosome instability are associated with a fragile site and are increased by defects in DNA replication and checkpoint controls in yeast. Genes Dev.

[B25] Pierce JC, Sauer B, Sternberg N (1992). A positive selection vector for cloning high molecular weight DNA by the bacteriophage P1 system: improved cloning efficacy. Proc Natl Acad Sci USA.

[B26] Shepherd NS, Pfrogner BD, Coulby JN, Ackerman SL, Vaidyanathan G, Sauer RH, Balkenhol TC, Sternberg N (1994). Preparation and screening of an arrayed human genomic library generated with the P1 cloning system. Proc Natl Acad Sci USA.

[B27] Lu C, Wang M, Greulich KM, Weier J, Weier HU, Liehr T (2008). Quantitative DNA fibre mapping. Springer Protocols: Fluorescence in situ hybridization (FISH) – Application Guide.

[B28] Barker D, Holm T, White R (1984). A locus on chromosome 11p with multiple restriction site polymorphisms. Am J Hum Genet.

[B29] Bepler G, Garcia-Blanco MA (1994). Three tumor-suppressor regions on chromosome 11p identified by high-resolution deletion mapping in human non-small-cell lung cancer. Proc Natl Acad Sci USA.

[B30] Neri A, Chang CC, Lombardi L, Salina M, Corradini P, Maiolo AT, Chaganti RS, Dalla-Favera R (1991). B cell lymphoma-associated chromosomal translocation involves candidate oncogene lyt-10, homologous to NF-kappa B p50. Cell.

[B31] O'Briant KC, Ali SY, Weier HU, Bepler G (1998). An 84-kilobase physical map and repeat polymorphisms of the gastrin/cholecystokinin brain receptor region at the junction of chromosome segments 11p15.4 and 15.5. Chromosome Res.

[B32] Mercurio F, Didonato J, Rosette C, Karin M (1992). Molecular cloning and characterization of a novel Rel/NF-kappa B family member displaying structural and functional homology to NF-kappa B p50/p105. DNA Cell Biol.

[B33] Bours V, Burd PR, Brown K, Villalobos J, Park S, Ryseck RP, Bravo R, Kelly K, Siebenlist U (1992). A novel mitogen-inducible gene product related to p50/p105-NF-kappa B participates in transactivation through a kappa B site. Mol Cell Biol.

[B34] Hsieh HB, Wang M, Lersch RA, Kim UJ, Weier HU (2000). Rational design of landmark probes for quantitative DNA fiber mapping (QDFM). Nucleic Acids Res.

[B35] Birnboim HC, Doly J (1979). A rapid alkaline extraction procedure for screening recombinant plasmid DNA. Nucleic Acids Res.

[B36] Weier HU, Rautenstrauss B, Liehr T (2002). Quantitative DNA fiber mapping. FISH Technology.

[B37] Weier HU (2001). DNA fiber mapping techniques for the assembly of high-resolution physical maps. J Histochem Cytochem.

[B38] Weier HU, Darzynkiewicz Z, Chrissman HA, Robinson JP (2001). Quantitative DNA fiber mapping. Methods in Cell Biology; Part B, Cytometry.

[B39] Weier HU, Chu LW (2006). Quantitative DNA fiber mapping in genome research and construction of physical maps. Methods Mol Biol.

[B40] Maniatis T, Fritsch EF, Sambrook J (1986). Molecular cloning: a laboratory manual.

[B41] Kimmerly W, Stultz K, Lewis S, Lewis K, Lustre V, Romero R, Benke J, Sun D, Shirley G, Martin C, Palazzolo M (1996). A P1-based physical map of the Drosophila euchromatic genome. Genome Res.

